# Investigating Automatic Vocal Rise Time Measurement Parameters and Multi-Corpus Vowel Onset Behavior with the Voice Onset Analysis Tool (VOAT)

**DOI:** 10.3390/bioengineering13080950

**Published:** 2026-08-21

**Authors:** Brian Stasak, John Holik, Duy Duong Nguyen, Tomás Arias-Vergara, Michael Döllinger, Cate Madill

**Affiliations:** 1 Sydney Voice Lab (Dr Liang Voice Program), Discipline of Speech Pathology, Faculty of Medicine and Health, University of Sydney, Camperdown, NSW 2006, Australia; john.holik@sydney.edu.au (J.H.); duong.nguyen@sydney.edu.au (D.D.N.); cate.madill@sydney.edu.au (C.M.); 2 Department of Otorhinolaryngology, School of Medicine and Pharmacy, Vietnam National University, Hanoi 100000, Vietnam; 3 Pattern Recognition Lab, Friedrich-Alexander-Universität Erlangen-Nürnberg (FAU), 91058 Erlangen, Bavaria, Germany; tomas.arias@fau.de; 4 Division of Phoniatrics and Paediatric Audiology, Department of Otorhinolaryngology Head & Neck Surgery, University Hospital Erlangen, Friedrich-Alexander-Universität Erlangen-Nürnberg (FAU), 91058 Erlangen, Bavaria, Germany; michael.doellinger@uk-erlangen.de

**Keywords:** acoustics, glottal attack, phonation, speech processing, voice disorders

## Abstract

Vocal rise time (VRT) acoustically measures from when vocal cord vibration begins to when phonation achieves a peak steady state. This acoustic-based study examines manual and automatic VRT measurements based on different vowel onset recordings (e.g., *hard*, *modal*, *soft*) from four datasets containing 146 adult Australian English-speaking females. Experiments test how the automatic Voice Onset Analysis Tool (VOAT) settings impact VRT measurement estimations. When compared to VOAT VRT baseline setting results, parameter adjustments using a Hilbert envelope 400 ms analysis segment with a low smoothing factor decreased VRT mean absolute error as low as 15.5 ms and 21.3 ms for *modal* and *soft* onsets, respectively. While the automated VOAT demonstrates objective repeatability with accelerated VRT extraction time advantages, still experiments herein show that the proposed adjusted settings are least accurate for *hard* onsets, whereby a mean absolute error as high as 71.0 ms is reported. Given three to five voice therapy sessions, females with voice disorders exhibited an 11% increase in VOAT VRT durations with reduced variation (6%), providing preliminary clinical evidence that this measure can assist in monitoring patients’ progress. This study reveals cross-corpora VRT norms per voice onset type, discusses VOAT parameter effects, and supports future automated VRT algorithm considerations.

## 1. Introduction

Vocal intensity is an important element that contributes to speech intelligibility, voice dynamics and perceptual quality [1]. Vocal rise time (VRT) is a temporal acoustic intensity measure based on vocal fold vibration onset during isolated vowel production. The VRT is measured by computing the duration of time from the vowel onset (start point) to the instance at which voicing intensity reaches its initial relatively peak steady amplitude (end point) [2,3]. VRT should not be confused with frequency stabilization time, which is based on fundamental frequency steadiness [4].

VRT characteristics have been measured by voice researchers to help categorize glottal attacks as *modal* (normal), *hard* (pressed, glottal), and *soft* (breathy, simultaneous easy) [3,4,5,6,7]. A *modal* voice onset involves voicing with moderate adductive tension, minimal turbulent noise, and a decrease in acoustic intensity energy as frequency rises (i.e., relatively linear descending spectral tilt) [8]. In the previous literature [2,4,7,8,9,10,11], *modal* voice recordings have been used to represent typical healthy control onset behavior, absent of voice disorders and/or trained extreme glottal manipulation. It should be noted that the term *modal* has been used interchangeably to describe vocal quality in both voice onset and post-onset behavior. A *hard* pressed-voice quality is characterized by laryngeal constriction (i.e., increased laryngeal muscle tension) with tight glottal adduction (i.e., relatively flatter spectral tilt compared to a *modal* voice), while a *soft* breathy-voice quality is characterized by wider glottal aperture with concomitant turbulent flow and lower longitudinal tension causing low vibratory rate (i.e., relatively steeper spectral tilt compared to a *modal* voice) [8]. A recent invasive visual-based study [2] has shown that in healthy speakers’ production of *soft* onsets, their glottis was pre-positioned in a spindle-like shape just before oscillation begins, while for *hard* onsets, their glottis shape was more hourglass-like with concomitant ventricular fold adduction. Further, visual analysis demonstrated that extreme breathiness was achieved via a significantly larger glottal area (i.e., greater separation between the vocal cords during adduction) [2].

Acoustic-based laryngeal manipulation studies [6,12] during sustained vowel production in different phonation types (i.e., intentionally altering voice production) for VRT analysis have reported variability in voice onset duration specifically during vocally trained performative constrained *hard* versus released *soft* false vocal fold activity. In *modal* phonation conditions, the number of cycles before vocal onset steady state can be variable even within the same speaker across multiple vowel utterances [2]. As a person’s expiratory flow increases, like often found in *soft* and/or *breathy* voice onset conditions, the number of steady-state cycles increase significantly (i.e., more time is required to reach a stable amplitude) [2]. Previous voice onset studies [2,3,4,5,6,10,11,12,13] have indicated that when compared to normal healthy *modal* onsets, speakers with pressed-voice *hard* onset have exhibited shorter VRT durations (<60 ms), while those with easy breathy-voice *soft* onsets have demonstrated longer VRT durations (>160 ms). However, in the past VRT literature [3,4,5,6,9,11,12,13,14,15,16], precisely allocated ranges per voice onset type have been difficult to clearly establish as these studies have reported varied onset type boundaries. Despite the agreed-upon *hard* to *soft* voice onset continuum, documented VRT range boundary imprecision is attributed to demographic speaker factors (e.g., age, gender, language), specific voice pathology, and how its measurements were derived [7,12,17,18,19]. For instance, females have a comparatively smaller sized larynx and typically shorter, stiffer vocal folds than males, resulting in naturally shorter *modal* voice onsets [19].

It has been established that voice disorders can adversely impact people’s voice onset function and acoustic characteristics [20]. Clinical voice studies [10,15,16] have also shown that individuals with voice disorders (e.g., laryngeal dystonia) produced abnormal phonation onset latencies, which resulted in auditory perceptually *hard* or *soft* qualities. In clinical settings, instructing voice-disordered patients on how to actively control their voice onset is an effective therapy approach, to reduce hyperadduction (as in the case of muscle tension voice disorders) [21]; or to increase true vocal fold closures (as in the case of Parkinson’s disease wherein maintaining loudness in spoken utterances may become difficult to achieve [10]). By learning and practicing physiological vocal attack onset via controlled, monitored vocal exercises, voice patients are often able to achieve auditory-perceptual improvements in voice quality and speech intelligibility without negative adverse effects of strain or discomfort [22].

Voice onset has previously been measured using auditory-perceptual (i.e., human expert judgement) [3,6,7,10], aerodynamic (e.g., phonatory airflow, volume/pressure) [7], physiologic (i.e., muscle movement) [7], visual (i.e., invasive high-speed laryngoscopy) [2,23] and acoustic (i.e., signal characteristic) measures [7,24]. Comprehensive systematic reviews [2,7] of VRT measures note a lack of evidence regarding each technique sensitivity, specificity and reliability to detect different voice onset types. Among the different voice onset measurement techniques reported in [7], the acoustic VRT measurement has the advantage over others because it only requires an audio voice recording, making it easily accessible outside of laboratory environments and less technically invasive. By using VRT to describe the voice onset during an initial baseline assessment and then tracking changes in patients’ VRT via multi-session voice therapy recordings, clinicians can obtain valuable information about their patients’ voice onset consistency, voice quality, association to specific voice disorder diagnoses, and remedial progress.

Despite the potential benefits to evaluating patients’ VRT behavior, its measurement has often relied on manual subjective audio–visual vowel onset data selection derived from digital waveforms and non-standardized analysis parameters, therefore making VRT results harder to comparatively interpret [7,22]. Previous VRT research using manual visual inspection [3,6,10] has included analogue/digital recorded audio voice waveform analysis using software such as Praat [25,26], or by perceptually grading voice recordings against idealized smooth onset envelopes [27]. While the manual visual-based method is currently the ‘gold standard’ for reporting VRT measurements, this approach comes with a few drawbacks because it must involve a skilled annotator who is familiar with vowel onset waveforms; still, it is not impervious to human error. Further, individually assessing VRT for each vowel onset recording is time consuming for large datasets, and introduces subjective variability in measurement accuracy given the potential for annotator fatigue, varied experience, quality of audio recordings, and customized visual analysis settings.

In recent studies [7,12,28,29,30,31], it has been proposed that automatically extracted VRT measurements computed from voice signal processing analysis software tools could help to minimize the need for subject manual data selection, decrease voice onset extraction timeframes, offer systematic analysis uniformity, support non-invasive voice health metrics, and aid in quickly flagging abnormal or sub-optimal voice onset habits in speakers that contribute to poorer voice quality. But there is still uncertainty towards how reliable automatically extracted VRT measurements are—and, importantly, whether certain voice onset types (e.g., *hard*, *modal*, *soft*) are more difficult to automatically measure accurately [12]. Thus far, to our knowledge, no voice study has compared manual versus automatic VRT batch extraction using multiple voice onset type datasets that include healthy, laryngeal manipulation, and voice-disordered speakers. Moreover, previous acoustic-based VRT studies [3,4,5,6,7,9,10,11,32,33] have separately evaluated no more than 71 female speakers at a time, often with minimal speaker vowel exemplar attempts (*n* = 1), therefore limiting the knowledge of normal intra-speaker onset behavior variability. A multi-corpora experimental investigation is needed to better establish the reliability of automated VRT extraction across a relatively larger set of speakers, establish voice onset norms, and establish new parameter setting guidelines for VRT extraction software.

This study evaluates two VRT measurement methods: (1) a subjective manual human one-by-one audio recording voice onset demarcation; and (2) an objective automatic Voice Onset Analysis Tool (VOAT) [12,28] which is the only open-source VRT voice analysis software that utilizes batch processing. Although VOAT VRT measures have been manually reported in studies [12,28], its level of automated precision involving different voice onset types has not yet been reported in published studies. This study is the first to evaluate its automatic VRT measurement accuracy. Manual human VRT measurements gathered during this study were used as a ‘gold standard’ quantitative baseline reference to compare measurements extracted from the new automated VOAT software. The aim of this study is to compare automated batch VRT VOAT extracted measurements to manual approach based on four different laboratory-quality voice datasets with 146 adult females containing multiple sustained /*a*/ onset type recordings. For the purposes of this study and to allow comparison with previous research, the terms *hard* (also known as ‘glottal attack’), *modal* (also known as both ‘glottal stroke’ and ‘easy’) and *soft* (also known as ‘breathy’) voice onsets are used. This study explores adult female VRT ranges per voice onset type, which voice onset type/s produce the largest automated VRT measurement error including voice onset start and end point precision, and explores VOAT software settings (e.g., analysis segment size, envelope type, smoothing factor).

## 2. Materials and Methods

### 2.1. Experimental Datasets

The three voice datasets evaluated in this study (see Table 1) were collected at The University of Sydney Voice Lab in Sydney, NSW, Australia. In all datasets, only adult female speakers (≥18 years) were included whilst male speakers were excluded due to their small sample size (*n* ≤ 4), limiting conclusive analytical statistical power and introducing additional confounding factors due to sexually dimorphic vocal tract length differences (i.e., gender-specificity influences the range norms for the VRT measurement [9,12,19]). For the University of Sydney Voice Cohort (USVC) [21], University of Sydney Laryngeal Manipulation Dataset (LMD) [12] and University of Sydney Vocal Onset (USVO) experimental datasets, inclusion criteria required that recorded participants had hearing within normal limits, Australian English as a first language, no known clinical voice condition/s, and were a non-smoker. For the Active Ingredients Dataset (AID) [32], which contained patients with voice disorders collected at a private Sydney voice clinic, the previously mentioned inclusions did not apply.

For all experimental datasets, recordings were conducted with clinically trained voice expert/s present in a quiet laboratory environment. The participants were recorded in a soundproof booth (USVC, LMD, USVO) or quiet room (AID) with a headset-mounted AKGC520 cardioid condenser microphone placed 5 cm and 45 degrees away from speakers’ mouths. All recordings were digitally captured at ≥44.1 kHz in mono-channel WAV format using digital multi-track recording software. An experimental dataset summary is shown in Table 1, with brief details for each discussed below. For more specific information concerning these voice onset behavior corpora and vocal training, refer to [12,21,32].

The University of Sydney Voice Cohort (USVC) dataset [21] contained 100 healthy young adult females with no known abnormal clinical voice conditions. This data was originally collected to study acoustic attributes in speakers with musical and non-musical backgrounds. Although the original USVC contained 115 females, 15 speakers had Voice Health Index scores beyond the norm threshold [21]; thus, they were excluded from this modal voice onset dataset. In the study herein, the USVC was utilized as a relatively large normative female control baseline to compare VRT ranges to the smaller sample sizes found in LMD and USVO datasets. Per USVC female speaker, 3 *modal* /*a*/ vowel onset recordings were utilized for VRT analysis. A total of 300 individual USVC recordings were evaluated for VRT analysis.

The Laryngeal Manipulation Dataset (LMD) [12] contained 5 healthy adult female participants with <1 year of training on voice onset types. Their voices had been collected in a previous study on the impact of laryngeal manipulation on voice quality [13,14], where participants were instructed on how to produce an /*a*/ vowel whilst altering their true vocal fold mass, false vocal fold activity, and larynx height by use of hands. These manipulations resulted in *modal*, thick constricted *hard* and thin breathy *soft* onset type voice qualities. The different LMD recorded voice onsets were verified using manual VRT visual–auditory inspection by three voice experts [12] (i.e., verified that acoustic attributes and VRT length were appropriate given the voice onset type recording). Per LMD female speaker, 1 *modal*, 4 *hard*, and 4 *soft* /*a*/ vowel onset recordings were utilized for VRT analysis. The LMD had a total of 45 individual recordings evaluated for VRT analysis.

The University of Sydney Vocal Onset (USVO) dataset consisted of 4 healthy middle-aged adult female voice experts (i.e., >10 years of training on voice onset types) who were specially trained in producing different types of voice onsets: *modal*, *hard*, and *soft*. USVO data was collected specifically to evaluate VOAT VRT software, and visual–auditory inspection per recording was validated by two voice experts. For each USVO female, 5 different /*a*/ vowels were recorded per *modal*, *hard*, and *soft* voice onset type. Therefore, 60 individual USVO recordings were utilized for VRT analysis.

The Active Ingredients Dataset (AID) [32] included 37 voice-disordered adult female patients with muscle tension dysphonia undergoing VoiceCraft^®^ Sob Voice Therapy [31]. The AID included progressive chronological therapy session timepoints recorded over the course of a year which included 3 /*a*/ vowel examples per session: (Session 1) 27 pre-therapy baseline patients; (Session 2) 37 post optimal phonation task training patients; (Session 3) 32 post sub-voice quality task training patients; (Session 4) 15 post sub-voice quality variation task training patients; and (Session 5) 17 post-negative practice task training patients. A total of 70% of AID patients completed 3 sessions, while 30% completed 4 to 5 sessions. From the pre-therapy baseline session to final post-negative practice sessions, improvement in patients’ perceptual and acoustic voice quality measurements were previously reported in Madill et al. [32]. Per AID female, the number sessions ranged from 3 to 5, with a total of 384 individual recordings utilized for VRT analysis.

### 2.2. Vocal Rise Time Software

To manually demarcate ‘gold standard’ voice onset VRT durations, Voice Onset Analysis Tool (VOAT) VRT software (version 1.0) [28] was used, which was developed by the Pattern Recognition Lab at Friedrich-Alexander University in Erlangen, Germany and the University of Sydney, Australia. The VOAT is a standalone GUI software based on Python code and it is freely available for download (see the Supplementary Materials section for link). The VOAT is the only speech signal processing software specifically designed for manually/automatically measuring VRT from recorded voice onsets (i.e., no state-of-the-art deep learning approach is currently available).

**Figure 1:**
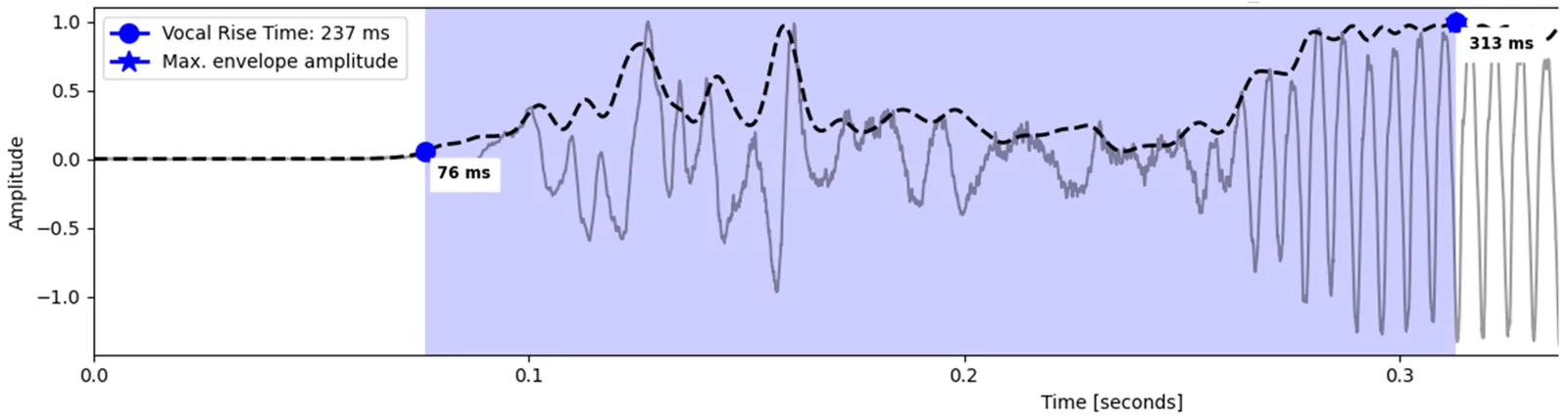
VOAT VRT software 400 ms window RMS envelope visual analysis output (shown by the black line) based on an LMD dataset adult female’s ‘breathy’ sustained /*a*/ vowel audio recording. The waveform (solid gray line) shows a vowel onset start point at 76 ms to its estimated stabilized peak rise end point at 313 ms. Thus, the automatically estimated VRT duration was 237 ms, which is indicative of a *soft* vocal onset range.

Shown in Figure 1, the VOAT facilitates visual manual VRT acoustic waveform analysis, which in a previous study [12] demonstrated ‘excellent’ manual inter-measurer reliability when using identical settings (≥0.99 inter-class correlation coefficient). VOAT VRT manual extraction requires human inspection to demarcate the approximate vowel onset start point and its onset peak amplitude end point via its graphical user interface waveform visual aid. The VOAT also supports a new automatic batch extraction option—meaning that it can quickly load, custom parameterize, and extract VRT measurements from multiple WAV recordings in a directory without requiring any human visual-based waveform demarcation analysis.

### 2.3. Vocal Rise Time Extraction Parameters

Manual VRT measurements were extracted from each of the experimental datasets shown in Table 1 by a voice research assistant trained in VRT measuring and using the VOAT software [28]. Per recording, a visual waveform inspection was conducted to manually calculate the onset-to-peak VRT measurement. This process was conducted in hour-long intervals with frequent breaks to minimize measurer fatigue. The manual approach, which included WAV file loading, visual start/end voice onset timepoint demarcation, and raw VRT measurement logging, took approximately 30 s per audio voice recording (i.e., an estimated continuous 6.5 h to complete VRT measurements for the four datasets in Table 1). For each of the experimental datasets, VRT measurements were also automatically extracted using the VOAT batch processing option, which took approximately 5 min.

For baseline manual and automatic VRT measurements, analysis settings were based on previous quiet laboratory-quality recording voice onset studies [3,12,27,28,31]. A default 400 ms analysis segment and root mean square (RMS) amplitude envelope with a smoothing factor {SF1} along with the voice activity detection setting ‘on’ was used for the baseline. In addition, a conservative 10% onset VRT start point threshold with 90% saturation end point cutoff setting was selected [12,27,33]. In non-laboratory environments (e.g., smart device, telephone), the threshold/saturation parameter options allow for more dynamic intensity scaling to counter undesirable background noise and/or uncalibrated microphone conditions which can mislead VRT measurement extraction software.

Additional VOAT parameter options were explored to procure VRT measurements closer to the manual method. The VRT analysis segment size setting can be adjusted for greater temporal resolution (100 ms to 500 ms), allowing for narrower or wider evaluation of voice onset-to-peak moments. In a previous study [12], manual VRT VOAT measurements were extracted using a 200 ms analysis segment setting. However, this study [12] showed no significant VRT class distinction between *hard* and *soft* voice onset types—strongly indicating that a longer analysis setting might have improved their reported estimations (i.e., other manual VRT voice onset studies [3,27,28,33] utilized a 400 ms analysis segment).

The VOAT also offered additional envelopes to the RMS, such as the peak amplitude envelope [12,34], which establishes the maxima voice peak based on a neighboring peak-to-peak analysis. But one disadvantage of the peak amplitude envelope is that it can generate incorrect VRT results if the peak maximum is outside of the search range or it is unrelated to voice energy (e.g., background noise, microphone clamor). Additionally, VOAT included the Hilbert envelope [28,35], which is a linear operation that acts as a filter and shifts the phase of all frequency components in a signal. The VOAT Hilbert transform was utilized to calculate the minimum-phase response derived from the acoustic-spectral voice recording analysis. Its transformation is filter-like and shifts the phases of *all* frequency input components by ±90°. When applied to the voice recording signal, the Hilbert transform results in an analytic version of its real and imaginary components [28]:

(1) s(t)=x(t)+iy(t)

where *s*(*t*) represents the analytic signal, *x*(*t*) denotes the real component (original acoustic signal), and *y*(*t*) is the imaginary component (Hilbert transform of *x*(*t*)). Thereafter, the amplitude envelope is computed as the magnitude of the derived analytic signal [28]:

(2) $$e(t)=\sqrt{x^{2}(t)-y^{2}(t)}$$

The resulting envelope *e*(*t*) is convolved with a Gaussian window. The Hilbert envelope results in a Gaussian window transformation that retains critical VRT temporal information and also helps to suppress short-term fluctuations in the original signal that can often be related to undesirable background noise. The VOAT includes a 0 to 5 envelope smoothing factor (SF) option, which adjust the aggressiveness of the voice signal amplitude contour by 20 ms increments ranging from 0 ms to 100 ms [28].

### 2.4. Experimental Setup and Performance Metrics

**Figure 2:**
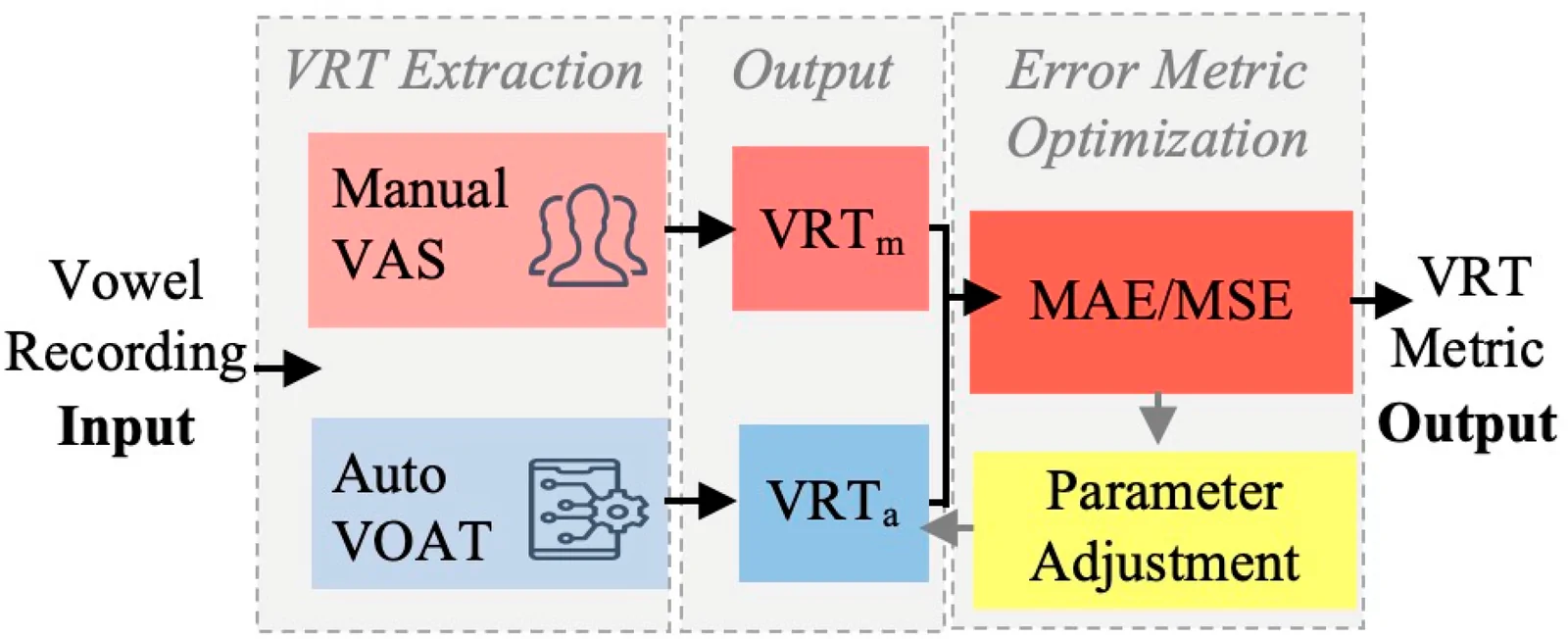
Experimental flowchart of manual versus automatic VOAT software VRT measurements. The error metric optimization process (gray arrows) included adjustment of VOAT VRT parameters to lower its VRT estimation error.

In Figure 2, the experimental setup initialized with the extraction of VRT measures via the manual demarcation and automatic VOAT software. Thereafter, the manual VRT measurements were then compared to the automated VOAT VRT measurements using a mean absolute error (MAE) (i.e., determination of the distance of similarity between these two measurements). The MAE is a commonly used performance metric during prediction/estimation analysis [36], and it is mathematically shown as follows:

(3) $$MAE=\frac{1}{n}\sum_{i=1}^{n}\left|y_{i}-{\hat{y}}_{i}\right|$$

where *n* is the total of VRT measurement observations and $\left|y_{i}-{\hat{y}}_{i}\right|$ is the absolute error (i.e., sum of absolute differences) between the ‘ground-truth’ manual VRT measurements and automatic VOAT VRT measurements. Therefore, the lower the MAE value, the closer the automated VOAT VRT measurement was to the manual human VRT measurements. The mean signed error (MSE) was also calculated to indicate whether the automatic VOAT VRT measurement results were overestimated or underestimated. MSE is calculated like in Formula (3), but it does not make use of absolute values. Therefore, if the automated VOAT VRT MSE result is ‘positive’, it is underestimated, while if the MSE result is ‘negative’, it is overestimated.

In addition to calculating MAE/MSE for the VOAT VRT length measurements, the precision of the estimated VRT voice onset start and end points were assessed using this same methodology—therefore, revealing if a particular VOAT parameter and/or onset type VRT resulted in more accurate VRT start and end point detection. The VRT start ($S_{i}-{\hat{S}}_{i}$) and end ($E_{i}-{\hat{E}}_{i}$) point MSE results provide greater insights towards where exactly the automated estimation error is occurring. For example, for a voice onset recording, an automated VOAT VRT measurement could obtain an identical value for two different parameters but differ with one having most of its estimation error contributed by its VRT start point, while the other demonstrating the bulk of its error attributed to its VRT end point.

In addition, paired Welch’s *t*-tests (*p*-value ≤ 0.05) with a *moderate-to-strong* Hedges’ effect size (*g* = ≥0.40) were used to establish if *modal* onset mean VRT measurements were statistically significant from *hard* or *soft* onsets. In Figure 2, VOAT VRT parameter variables (e.g., segment size, envelope, smoothing factor) were systematically tested to try to secure a new understanding towards more universal adjusted VOAT VRT software parameters, with the aim to explore how to lower MAE for *all* four datasets and different voice onset types.

## 3. Results

### 3.1. Baseline Parameters

The aim of this study was to investigate estimated VRT measurements derived from manual human versus automatic VOAT software using vowel onset datasets that included different voice onset types (e.g., *modal*, *hard*, *soft*). For the USVC, LMD, and USVO datasets, manual *modal* VRT measurements averaged 61 ms to 166 ms. Previous studies [3,4,5,6,10,15,16] have reported healthy normal *modal* VRT ranges between approximately 60 ms to 160 ms. Therefore, apart from the USVO dataset (166.1 ms), which was slightly higher than the norm, the manual measured VRT *modal* onset results herein concurred with the aforementioned literature. As shown in Table 2, the manual VRT measurements for the *hard* onset were in the higher range for LMD (57.7 ms) and USVO (73.2 ms) datasets. But manual VRT measurements for the *soft* onset were significantly different than *modal* onset for LMD (218.7 ms) and USVO (261.8 ms), thus giving clear indication of ‘breathy-like’ voice qualities associated with a longer and/or erratic VRT peak slope stability (see previous Figure 1).

The Table 2 baseline shows that a larger MAE was reported for the *soft* onset per dataset and therefore it was the least accurately automatically measured voice onset type. The largest baseline VRT MAE difference was reported in the LMD *soft* onset (116.3 ms), whereby the automatic VOAT generated an VRT average of 166.1 ms when compared to the manual VRT average of 218.7 ms. The baseline automatic VOAT LMD *modal* VRT average (26.4 ms) was incorrectly estimated in the *hard* onset range and much lower than the report manual average (61.0 ms). Further, the automatic LMD *hard* onset average (70.4 ms) was incorrectly in the *modal* range. For the AID manual VRT measurements, as therapy sessions increased, the average VRT increased (i.e., approximately a 21% increase from Session 1 to 5).

It is believed that the reason why the manual AID average session VRT values stay within the *modal* range is in part due to variation in patient voice disorder severities and voice quality factors (e.g., breathy, pressed), which at opposite far-ends would average together towards the *modal* normative range. For example, across Sessions 1 through 5, the AID manual VRT generated a 68 ms standard deviation and minimum to maximum VRT range of 12 ms to 360 ms. The AID Session 1 to 5 baseline automatic VRT average (58.3 ms) was nearly twice as low as its manual session average (109.5 ms). Moreover, the AID session automatic VOAT VRT baseline MAE was almost equal to the per-session VRT averages, which demonstrated a large degree of error for the baseline VRT VOAT settings.

In Table 2, in the USVC dataset, given the fairly large number of females (*n* = 100) and three speaker recordings per speaker, its baseline VRT intra-speaker standard deviations reported for the *modal* voice onset type were relatively stable (~24 ms to ~28 ms) for both manual and automated VRT methods. On the contrary, the USVO dataset demonstrated much higher *modal* voice onset manual/automatic baseline VRT intra-speaker standard deviations (~59 ms to ~66 ms) than the USVC. While the USVO only contained four females (i.e., limited a sample size), its larger intra-speaker standard deviation is unusual given that this dataset included speakers who were voice experts with more than 10 years of voice onset experience. It was anticipated that the USVO voice experts were the most likely to produce the lowest *modal* voice onset VRT intra-speaker standard deviation reported. This is the first study to demonstrate that even for well-trained female voice experts familiar with different voice onset types, including specific production of a healthy *modal* voice onset type, VRT measurements can still widely vary per individual speaker across multiple recorded attempts.

For the LMD and USVO datasets, the *hard* voice onset type baseline VRT intra-speaker standard deviations were lowest (~41 ms to ~48 ms) for the manual method. Further, the soft voice onset VRT intra-speaker standard deviation was largest (120 ms) for the manual method. In Table 2, the large value for the manual *soft* onset intra-speaker standard deviation was expected because the *soft* onset has the widest range (e.g., ~160 ms to ~400 ms). In their attempt to produce healthy *modal* onsets, AID dataset females with voice disorders generally struggled to produce consistent vowels based on manual VRT intra-speaker standard deviation results (i.e., most sessions between ~40 ms and ~50 ms and higher than the *modal* in the USVC dataset). In Table 2, AID dataset baseline VRT intra-speaker standard deviations across different therapy sessions were lower for the automatic than the manual method, therefore mirroring the relatively large VRT MAE using automated method with baseline parameter settings.

The MSE results reported in Table 2 demonstrated that the baseline VOAT settings frequently underestimated the VRT length, as only the LMD and USVO *hard* voice onsets were overestimated. For the baseline VOAT results, *soft* voice onsets were among the highest MSE results reported (e.g., LMD = 115.8 ms, USVO = 108.5 ms). Additionally, according to the MSE results in Table 2, the AID patients’ VRT measurements were often overestimated (36.7 ms to 65.7 ms). More details regarding whether the general baseline MSE VRT underestimation error was due to inaccurate VOAT VRT start or end point detection is further discussed in Section 3.2.

The automatic VOAT analysis segment size parameter demonstrated a considerable impact on how well VRT was estimated for specific voice onset types. For example, when the 200 ms VOAT evaluative window setting was employed, it was better at estimating *hard* than *soft* onsets because *hard* onsets are typically <60 ms in duration and still within the evaluative analysis segment size of 200 ms. However, *soft* voice onsets often surpass a set analysis segment size of 200 ms, which, given any voice onset > 200 ms, will be inaccurately underestimated. Therefore, for automatic VOAT VRT it was proposed to use a larger analysis segment size setting of 400 ms—therefore, ensuring that *hard*, *modal* and *soft* onsets ranges could be measured. It is noted, though, that the automatic VOAT 400 ms analysis segment setting adversely impacted *hard* onset VRT precision (i.e., this a bias related to analysis segment size). A future VOAT algorithm update will include automated voice signal typing (i.e., similar to [37]), rather than a fixed setting, to help pre-determine the VOAT analysis segment size parameter per voice recording.

**Figure 3:**
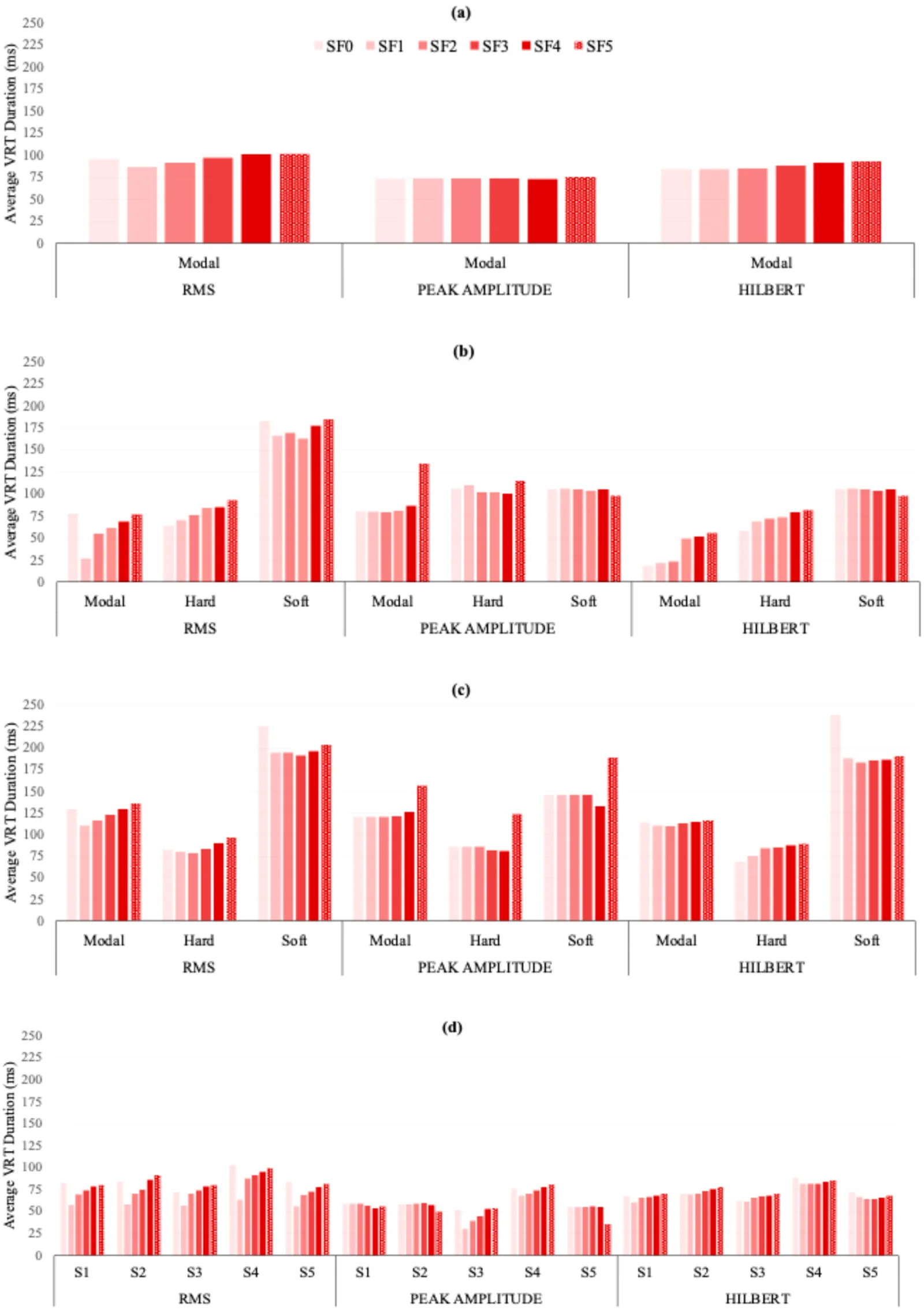
Per dataset ((**a**) USVC, (**b**) LMD, (**c**) USVO, and (**d**) AID), average automatic VOAT VRT durations given incremental envelope smoothing factor parameters {SF0 to SF5} for 3 different signal envelopes (e.g., RMS, peak amplitude, Hilbert). Baseline VOAT setting results are shown with texture.

In Figure 3, baseline incremental adjustment experiments of automatic VOAT smoothing factor parameters across the three different envelope types demonstrated that larger settings conversely increased average VRT duration estimates. For example, smoothing factor (SF) setting experiments based on the USVC (Figure 3a) showed VRT duration gains of 17% when going from {SF0–SF1} to {SF4–SF5} setting extremes (i.e., SF0 is no smoothing factor applied). In particular, the largest VRT duration trend gains were observed when using the RMS and Hilbert envelopes, while the peak amplitude envelope was less influenced by smoothing settings. Similarly, the LMD (Figure 3b) average VRT durations also increased up to 198% given RMS and Hilbert smoothing factor extremes {SF0–SF1} to {SF4–SF5}. This smoothing-factor-related average VRT duration increase trend was also observed with the USVO (Figure 3c) and AID (Figure 3d). The explanation for this phenomenon relates to its function, whereby a low smoothing factor {SF1} value results in an amplitude envelope more closely fitted to the contour of the original signal, and a high smoothing factor value results in an amplitude envelope that describes the broad shape of the signal [26]. Therefore, in a clean recording environment, the advantage of using a low smoothing factor VOAT setting is that it more accurately identifies the vocal onset start point contours. Based on high-quality laboratory voice recordings, Figure 3 results demonstrate that smoothing factor settings {SF0, SF5} were the least stable and often produced more unreliable VRT estimates.

The VRT analysis, which was originally defined in [33] (p. 303), stated that it is the “… *time needed for an envelope of the acoustic signal to go from 10% to 90% of the maximum amplitude…*”. But this evaluation percentage is heavily dependent on the VOAT analysis segment size parameter, which again needs to be long enough (~400 ms) to properly measure *soft* voice onsets. In a related hybrid surface electromyography voice onset study by Stepp et al. [33], a 20% to 80% maximum amplitude analysis window size parameter was used. However, Stepp et al. [33], like other many studies before [3,4,5,6,10,12], did not systematically test analysis window sizes across different onset types from a relatively large number of speakers and onset types. In experiments herein, automatic VOAT VRT results were extracted using {10%, 5%, 1%, 0%} threshold and {90%, 95%, 99%, 100%} saturation combination settings. Based on these automatic VOAT VRT estimates, the 5% threshold demonstrated the best voice onset start point fit, with a 100% end point saturation setting more effectively measuring *soft* voice onsets (i.e., end point saturation ≤ 95% struggled to accurately place these in the >160 ms *soft* voice onset range). Preliminary investigation results based on VOAT setting experiments herein indicated that a higher threshold saturation (≤95%) and/or analysis segment size (~400 ms) is better for measuring *soft* voice onsets, while lower saturation setting (~90%) and/or a lower analysis segment size (~200 ms) is more advantageous for measuring *hard* voice onsets. However, further experiments on more varied speaker demographics, onset recording types, and recording environments are needed to validate the robustness of VOAT threshold/saturation settings and what threshold/saturation settings are most appropriate given these different factors.

### 3.2. Adjusted Parameters

Table 3 shows automatic VOAT VRT results utilizing combined experimental adjusted parameters. The use of Hilbert envelope with 400 ms analysis segment and wider voice onset start and end point threshold/saturation ranges of 5% to 100% resulted in effectively decreasing the VRT MAE for many datasets when compared to baseline settings. Shown in Table 3, when compared to the baseline results (shown in Table 2), adjusted automatic VOAT VRT results demonstrate MAE improvements for eight out of twelve of the dataset voice onset types, especially the *modal* and *soft* onsets. For example, adjusted settings for LMD and USVO produced lower VRT MAE for *modal* (15.5 ms; 18.2 ms) and *soft* (21.3 ms; 30.8 ms) onsets, when compared to the baseline MAE (34.6 ms; 55.2 ms, 108.5 ms; 116.3 ms).

Based on Table 3, further analysis of the adjusted automatic VRT results demonstrated that two of the four *hard* onset subtypes produced in the LMD dataset (e.g., constricted thick low-larynx and constricted thick normal-larynx) were responsible for the majority of automatic VRT estimation error. In fact, without these two unusual *hard* onset voice recording exemplars (i.e., keeping only the constricted thin low-larynx and constricted thin normal-larynx), the adjusted automatic VOAT VRT MAE for *hard* onset was further reduced to just 5.2 ms (i.e., 63.9 ms MAE without constricted thick low-larynx; 34.4 MAE without constricted thick normal-larynx). These two *hard* onset productions involve vocal muscular activation that counter each other—indeed, an odd movement typically only observed in professionally trained speakers and rarely exhibited by voice disorder patients. Nonetheless, this is the first time in the voice processing onset literature that experimental automatic VRT results have demonstrated difficulty in accurately measuring *hard* onset constricted thick low-larynx/normal-larynx female vowel recordings.

In Table 3, for the USVC dataset, the *modal* voice onset automatic adjusted VRT intra-speaker standard deviation (~26 ms) reported was nearly identical to the manual approach (~24 ms). In addition, for the LMD and USVO datasets, the automatic optimization of the VRT intra-speaker standard deviation for *soft* voice onsets (~128 ms) also were closer to the manual results (~120 ms) than the baseline automatic approach. In Table 3, even though the automatic adjusted VRT intra-speaker standard deviation for the AID dataset was generally larger per session, its VRT MAE results were still lower than the baseline reported in Table 2.

**Figure 4:**
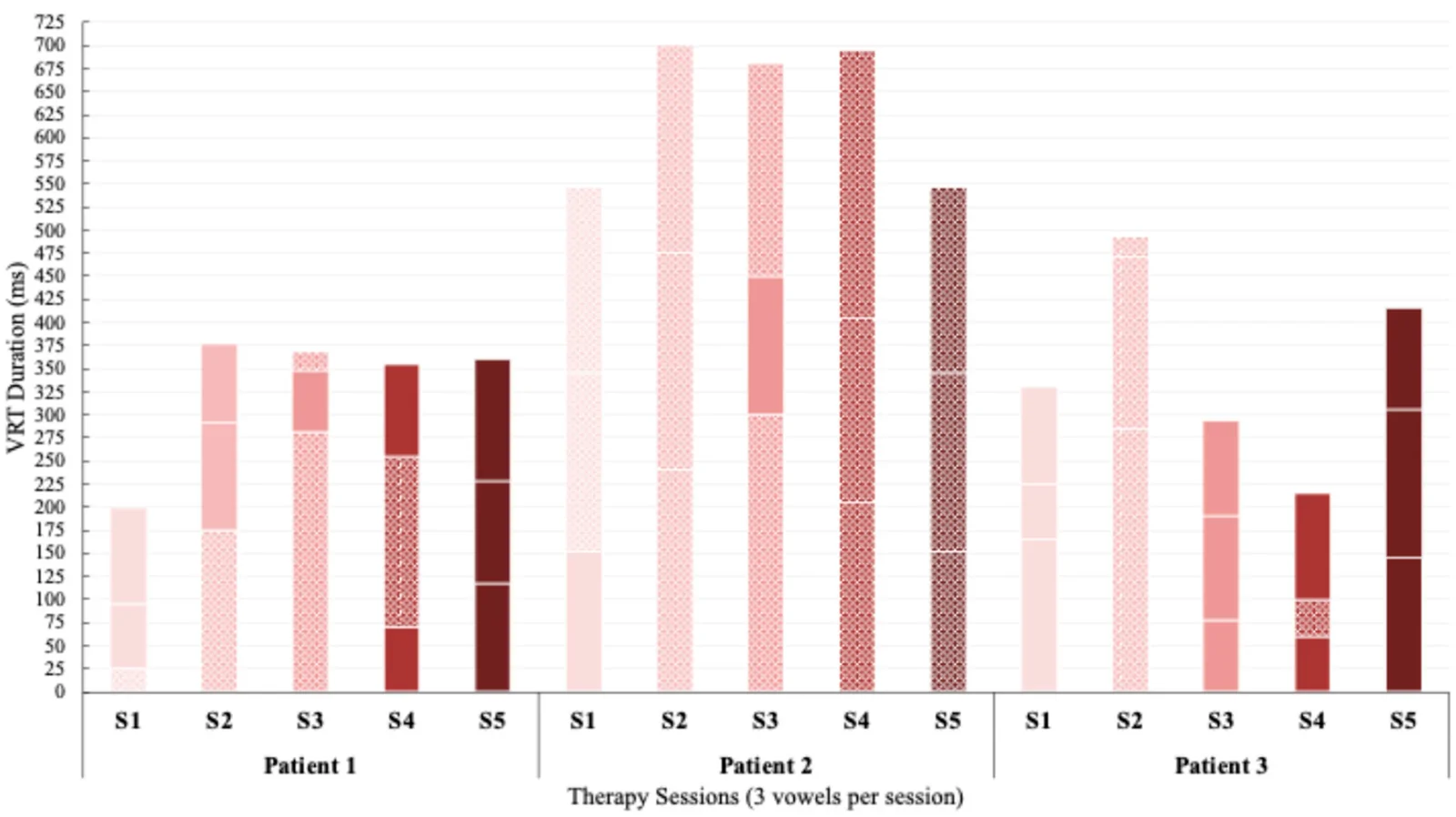
Manual VRT duration comparison of 3 female muscle tension dysphonia patients who completed all 5 therapy sessions {S1–S5}. The bar segmentations per session indicate the duration of the VRT per 3 vowel exemplars. Solid shaded bars indicate VRT duration within 60 ms–160 ms *modal* voice onset range (i.e., normal/healthy), while textured bars indicate VRT duration in abnormal *hard* or *soft* voice onset ranges.

Collectively, adjusted automatic VOAT VRT measurements demonstrated relatively lower average MAE (43.8 ms average) across *all* datasets when compared to its baseline MAE (60.5 ms average). However, the AID recordings presented automated VOAT VRT measuring imprecision, likely due to the complex nature of muscle tension voice disorders on voice quality acoustic onset changes related to laryngeal manipulation therapy. Generally, by Session 5, as shown in later Figure 4, patients demonstrated greater consistency in their habitual vowel onsets (i.e., VRT durations nearly equal per attempt and closer to the healthy *modal* range). In Table 3, according to the MSE results, the adjusted VOAT parameter consistently overestimated the VRT measurement for every dataset and voice onset type.

**Table 3:** Adjusted vocal rise time (VRT) duration averages, mean absolute error (MAE), and mean signed error (MSE) based on manual versus automatic adjusted VOAT method (e.g., 400 ms analysis window, Hilbert envelope, smoothing factor {SF1}, 5–100% amplitude saturation). *Hard* and *soft* voice onset type average VRT duration results that were significantly statistically different from the *modal* type based on Welch’s *t*-tests (*p* ≤ 0.05) and Hedges’ effect size (*g* ≥ 0.40) are indicated by an *. The bold MAE values indicate an improvement in using the adjusted VOAT settings over the baseline standard settings results that were reported in Table 2. Average intra-speaker VRT standard deviation is shown in gray (±).

Voice Datasets	Onset Type/s or Session Number	Manual VRT Average (ms)	Automatic Adjusted VRT Average (ms)	VOAT VRT MAE [MSE] (ms)
**USVC**	*Modal {60 to 160}*	97.6 ±24.4	163.5 ±26.1	70.7 [−65.5]
**LMD**	*Modal {60 to 160}* *Hard {<60}* *Soft {>160}*	61.0 ~ 57.7 ±48.0 218.7 * ±120.0	73.2 ~ 127.3 * ±40.0 222.4 * ±127.9	**18.2 [−10.2]** 71.0 [−69.6] **21.3 [−0.58]**
**USVO**	*Modal {60 to 160}* *Hard {<60}* *Soft {>160}*	166.1 ±59.4 73.2 * ±40.5 261.8 * ±70.5	181.5 ±42.4 143.3 * ±64.6 266.6 * ±66.7	**15.5 [−15.5]** 70.4 [−70.1] **30.8 [−4.6]**
**AID**	*Session 1* *Session 2* *Session 3* *Session 4* *Session 5*	94.3 ±38.3 103.6 ±38.5 106.1 ±49.2 129.2 ±48.9 114.2 ±28.5	136.5 ±73.3 145.3 ±55.9 132.8 ±52.4 184.1 ±75.3 153.8 ±65.6	52.7 [−42.6] **44.4 [−41.6]** **27.8 [−26.6]** **55.1 [−54.9]** **47.2 [−39.6]**

For all experimental datasets herein, the baseline versus adjusted VOAT parameter VRT start point MSE averages were 8.31 ms and 1.55 ms, respectively. Moreover, the baseline and adjusted VOAT parameter VRT end point MSE averages were 20.36 and 39.0 ms, respectively. The VRT start point MSE comparative results demonstrate that the VOAT given either parameter is still relatively accurate at identifying where the voice onset occurs in laboratory-quality data. Moreover, in Table 4, it is highlighted that the precision of the VOAT VRT start time retained fairly accurate MSE values for the AID sessions, despite it containing patients with ongoing clinical voice disorders. Results show that the VOAT voice onset (start point) 5% threshold setting utilized during the adjusted parameters provided slightly better VRT start point precision than the 10% baseline setting.

Also, Table 4 clearly indicates that the largest degree of VOAT VRT estimation error is attributed to its ability to accurately locate the moment of voice offset peak stability (end point). The adjusted parameter results demonstrate that even in a quiet laboratory environment, a VOAT saturation setting of 100% is perhaps too aggressive, generating an overestimated MSE especially for *modal* (USVC = −58.4, LMD = −46.4) and *hard* voice onsets (LMD = −69.6 ms, USVO = −70.1 ms) and AID voice-disordered patients (−26.6 ms to −54.9 ms). Despite the baseline or adjusted VOAT parameters, the results in Table 4 show that VOAT struggles to accurately find the offset peak across a wider number of speakers and voice onset types, with experimental MSE ranging from 87.1 ms to −66.4 ms, thus providing an indication of where the VOAT peak detection algorithm could be further improved upon while also providing its users with a degree of caution in terms of its VRT end point detection accuracy. Again, this is the first time in the literature that the VOAT performance in terms of automated VRT estimation accuracy and start/end point precision has been evaluated.

**Table 4:** VOAT mean signed error (MSE) automatic baseline and adjusted parameter VRT start/end point estimations. With regard to locating voice onset time point precision, a positive value indicates a VRT underestimation, while a negative value reflects a VRT overestimation.

Voice Datasets	Onset Type/s or Session Number	Baseline VRT MSE (ms)		Adjusted VRT MSE (ms)	
		**Start**	**End**	**Start**	**End**
**USVC**	*Modal {60 to 160}*	10.8	21.4	7.2	−58.4
**LMD**	*Modal {60 to 160}* *Hard {<60}* *Soft {>160}*	6.7	−28.1	2.4	−46.4
		10.1	−55.6	−2.0	−57.5
		−2.5	−15.4	0.2	−24.8
**USVO**	*Modal {60 to 160}* *Hard {<60}* *Soft {>160}*	6.4	61.6	1.5	−14.0
		11.4	−1.6	3.7	−66.4
		21.4	87.1	−11.5	−16.1
**AID**	*Session 1* *Session 2* *Session 3* *Session 4* *Session 5*	6.1	31.0	−0.8	−41.3
		5.9	38.5	3.2	−36.2
		10.9	53.5	7.2	−20.0
		13.6	5.9	11.9	−42.7
		−1.0	46.1	−4.4	−44.2

### 3.3. Clinical Multi-Session Voice Patient Analysis

A VOAT VRT length case study analysis of three AID voice disorder patients who completed all five clinical therapy sessions is shown in Figure 4. Manual VRT results herein provide an indication of greater onset stability with increased number of voice therapy sessions and completion of the VoiceCraft^®^ Sob Therapy program. For example, the VRT standard deviation for these AID patients in pre-therapy Session 1 was considerably higher (39.7 ms) than later in post-therapy Session 5 (21.7 ms). Moreover, Patient 1 who had an initial pre-therapy session *hard* ‘strained’ voice onset demonstrated a noticeable 80.2% increase in VRT duration by Session 5, while Patient 2 often exhibited a *soft* ‘breathy’ voice onset throughout the sessions. While Patient 3 was not as extreme as the other patients in terms of VRT lengths, her results in Figure 4 show a more balanced convergence of VRT lengths by Session 5.

## 4. Discussion

Manually selecting VRT start to end timepoints based on a waveform for each individual recording requires training, time, and expert judgement. Results herein demonstrate that the automatic VOAT software can quickly generate VRT results when compared to a more onerous manual approach. In some voice onset type dataset instances, such as *modal*, it can automatically generate VRT MAE results within 15.5 ms of traditional manual measurements. But results reported herein also show that a degree of caution should be taken when utilizing the automated VOAT VRT outputs. It is recommended to have a subset of voice recordings in which the VRT measurements are manually verified to help re-affirm that the VOAT batch extraction parameter settings are appropriate, especially given what types of specific voice onset/s are analyzed.

Adjusted VOAT parameters using a Hilbert envelope (i.e., located onset time point more precisely) with a 5% threshold and 100% saturation setting (i.e., captured slow peak accent often found in soft onsets) produced VRT measurements that were more accurate across different test datasets in this study. VOAT results herein show that even a high 90% saturation amplitude level parameter threshold was not high enough to capture *soft* voice onsets VRT measurements and often generated misleading VRT calculations during automatic extraction. For quiet laboratory headset microphone audio recording conditions, a VOAT saturation threshold setting of ≥95% is recommended for *modal* and/or *soft* voice onset types—although this will have be further tested through experimentation involving a greater number of diverse speakers.

**Figure 5:**
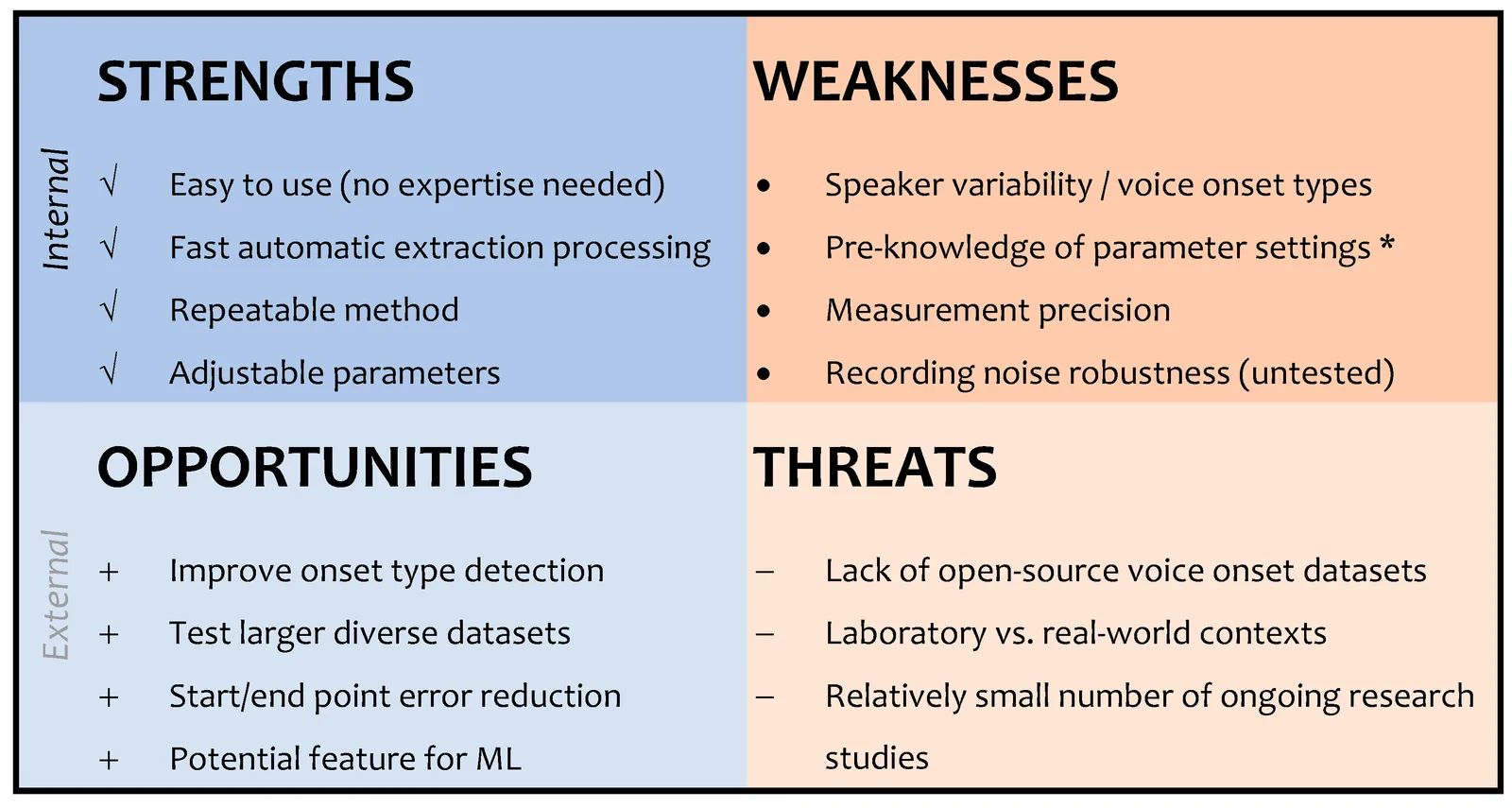
SWOT diagram highlighting the current advantages/disadvantages of the open-source automatic VOAT VRT tool. The * indicates that this study herein contributes to providing its users with a better understanding of how its measurements may be influenced by different VOAT parameter setting combinations and recorded voice onset types (e.g., hard, modal, soft).

A key motivation behind this study was to evaluate and to inform potential VOAT users on how different parameters influence VRT results, and, further, to provide guidance depending on what voice onset types are evaluated. To our knowledge, the VOAT is currently the only open-source software providing automated individual and batch VRT extraction analysis. In Figure 5, based on the findings herein, a SWOT analysis summary is provided regarding the VOAT VRT extraction tool. A major strength of the VOAT is that a user with little VRT experience can quickly extract measurements. Further, the VOAT provides a method for repeatable extraction, unlike manual methods, which can vary depending on expertise. Also, the VOAT contains adjustable parameters which can help to improve VRT precision for specific onset types.

As indicated by the results found in Section 3.1, Section 3.2 and Section 3.3 and shown in Figure 5, the automated VOAT software still presents weaknesses, some of which can be semi-addressed if there is pre-knowledge of what type of voice onset was recorded. For instance, if there are several hundred recorded examples of speakers with breathy-like voices (e.g., *soft*, *simultaneous easy*), a VOAT user can adjust the analysis window size to 400 ms to more accurately capture voice onset measurements that reflect slower rising stability peaks requiring a longer analysis window. While results presented in this study only include studio-quality recordings, more research using the VOAT on non-laboratory-collected voice data across different devices is warranted—and could further demonstrate its real-world usefulness in remote medical therapy and monitoring settings.

There are opportunities to study voice onset behavior using the VOAT with more diverse speakers (e.g., non-adults, males, non-English languages). Those future VRT experiments would provide more information regarding intra-/inter-speaker voice onset variability per onset type. Further larger-scale VRT extraction could support machine learning modeling applications such as automatic voice disorder identification. It is acknowledged that the previous literature has depended on relatively small datasets, none of which are open source. Thus, there is a considerable experimental data limitation with regard to voice onset type examples and clinically validated datasets. Future voice onset datasets would support the improvement of our understanding of VRT behavior and further validation of automated VRT algorithms and tools, such as the VOAT software.

Findings in the present study were limited in that only adult female recordings were evaluated. While the total number of females evaluated was larger than previous studies mentioned in the Introduction, more research is needed to examine VRT in adult male and non-adult speaker populations. Further, the VRT results reported in this study evaluated sustained /*a*/ vowels. Therefore, it is less known how onset behavior and VRT measurements compare during natural speech and/or other sustained vowel tasks. Also, while this study included an examination of voice onset behavior in individuals with voice disorders, greater investigations across different voice disorder diagnoses (e.g., functional, organic, psychogenic, neurological) are needed to help determine whether the VRT metric is appropriate for measuring and/or monitoring other types of voice pathologies.

This VOAT VRT experimentation was limited to adult native English female speakers’ /*a*/ vowel. Thus, experiments herein did not address socio-cultural linguistic (e.g., phonetic, language/dialect, co-articulation) and other demographic factors (e.g., gender, age, height, years vocal training, specific disorder type/s). While this study reports changes in adult female pathological voice patients undergoing clinical voice therapy, its number of patients is still somewhat small compared to other phonological phenomena studies [7]. While the number of total speakers evaluated in this study is among the largest specifically concerning acoustic-based VRT analysis, it is proposed that larger-scale ‘big data’ speaker analysis including thousands of speakers with diverse backgrounds would be beneficial to better understand vocal rise time onset behavior and how demographic factors may impact speaker onset type range norms.

In the future, an expanded VRT should be planned which would involve an investigation of this new VOAT software on a greater number of datasets containing many different voice disorders with broader speaker demographics (e.g., age ranges, gender, multiple languages), varied audio quality (e.g., microphone types, recording devices) and an updated version including additional VRT-related acoustic signal features. Results herein indicate that the analysis segment size parameter had a divergent-like impact on accuracy for extreme *hard* versus *soft* voice onset types, which should be addressed in future VOAT algorithm development. On a per-voice-recording basis, it is believed that automated signal typing for ‘breathy’ versus ‘pressed’ voice quality could help pre-determine an appropriate VOAT analysis window size, a smaller (<100 ms) or larger (>200 ms), respectively. However, currently, if the speaker/s signal type is known before automated VOAT processing, this speaker signal typing information can be leveraged to pre-select a more appropriate VOAT analysis size setting—effectively improving the precision of the automated VRT measurement, as discussed and shown in Section 3.

## 5. Conclusions

This experimental study compared manual human to automated VOAT software VRT measurements across multiple datasets. Experimental adjusted VOAT parameter setting results demonstrated automatic VRT estimation improvements in three out of four different voice onset datasets; moreover, they showed improved MAE precision over baseline VOAT settings, especially for *modal* and *soft* voice onset types. VOAT VRT baseline and adjusted parameter multiple database experiments showed more precision with VRT onset start point than the end point, where a greater degree of error occurs. This indicates that algorithmic improvements in the estimation of the voice onset peak stability (end point) are needed. Additional analysis demonstrated that for voice therapeutic application, the manual and/or automatic VOAT software can be used to non-invasively monitor changes in VRT behavior associated with increased voice therapy sessions.

## Figures and Tables

**Table 1:** Summary of the four datasets analyzed for vocal rise time (VRT) experiments. Per dataset, the individual /*a*/ vowel audio recordings evaluated were from adult female speakers.

Voice Datasets	Voice Onset Types	# of Recordings	# of Speakers	Average Age (yrs)
**USVC**	*Modal*	300	100	23.1 ± 3.8
**LMD**	*Modal*, *Hard*, *Soft*	45	5	27.5 ± 5.5
**USVO**	*Modal*, *Hard*, *Soft*	60	4	40.4 ± 16.8
**AID**	*Disordered (5 Sessions)*	384	37	32.9 ± 10.7

**Table 2:** Baseline vocal rise time (VRT) duration averages, mean absolute error (MAE), and mean signed error (MSE) based on manual versus automatic VOAT method. Results were derived from adult female /*a*/ vowel recordings and utilizing the default 400 ms analysis window, RMS envelope, smoothing factor {SF1}, and 10–90% amplitude saturation parameters suggested previously in [12]. *Hard* and *soft* voice onset type average VRT duration results that were significantly statistically different from the *modal* type based on Welch’s *t*-tests (*p* ≤ 0.05) and Hedges’ effect size (*g* ≥ 0.40) are indicated by an *. Average intra-speaker VRT standard deviation is shown in gray (±). The LMD *modal* voice onset type only contained 1 recording; hence, its missing VRT standard deviation results.

Voice Datasets	Onset Type/s or Session Number	Manual VRT Average (ms)	Automatic Baseline VRT Average (ms)	VOAT VRT MAE [MSE] (ms)
**USVC**	*Modal {60 to 160}*	97.6 ±24.4	87.0 ±27.9	41.6 [10.4]
**LMD**	*Modal {60 to 160}* *Hard {<60}* *Soft {>160}*	61.0 ~ 57.7 ±48.0 218.7 * ±120.0	26.4 ~ 70.4 * ±76.2 166.1 * ±76.4	34.6 [34.6] 49.1 [−12.8] 116.3 [115.8]
**USVO**	*Modal {60 to 160}* *Hard {<60}* *Soft {>160}*	166.1 ±59.4 73.2 * ±40.5 261.8 * ±70.5	110.8 ±66.1 86.1 * ±85.4 153.3 * ±91.0	55.2 [55.2] 46.8 [−12.9] 108.5 [108.5]
**AID**	*Session 1* *Session 2* *Session 3* *Session 4* *Session 5*	94.3 ±38.3 103.6 ±38.5 106.1 ±49.6 129.2 ±48.9 114.2 ±29.5	57.7 ±24.4 57.9 ±22.6 56.6 ±19.6 63.5 ±20.0 55.6 ±17.8	44.3 [36.7] 50.7 [45.7] 53.8 [49.5] 67.1 [65.7] 58.5 [58.5]

## Data Availability

The datasets are unavailable due to participant privacy restrictions.

## Supplementary Materials

The VOAT vocal rise time analysis software can be publicly downloaded at: http://www.github.com/TAriasVergara/VOAT_Project/tree/main (accessed on 25 July 2025).

## Abbreviations

The following abbreviations are used in this manuscript:

AID	Active Ingredients Dataset
LMD	Laryngeal Manipulation Dataset
MAE	Mean Absolute Error
USVC	University of Sydney Voice Cohort Dataset
USVO	University of Sydney Vocal Onset Dataset
VRT	Vocal Rise Time
VOAT	Voice Onset Analysis Tool
